# Spread of injectate during the transversalis fascia plane block: a preliminary anatomical investigation

**DOI:** 10.1186/s44158-026-00375-x

**Published:** 2026-03-18

**Authors:** Bahadir Ciftci, Serkan Tulgar, Aybegüm Fazlioglu, Bora Bilal, Selcuk Alver, Izzet Alatli, Ali Osman Korkmaz, Bayram Ufuk Sakul, Alessandro De Cassai, Ali Ahiskalioglu

**Affiliations:** 1 Department of Anatomy, Istanbul Medipol University, Istanbul, Turkey; 2 Department of Anesthesiology and Reanimation, Istanbul Medipol University, Istanbul, Turkey; 3 Bahçeşehir University Hospital Medical Park Göztepe, Department of Anesthesiology and Reanimation, Istanbul, Turkey; 4 Clinical Anatomy PhD Program, Graduate School of Health Sciences, Istanbul Medipol University, Istanbul, Turkey; 5 Department of Anesthesiology and Reanimation, Istinye University Liv Hospital Topkapı, Istanbul, Turkey; 6 Biruni University Hospital, Anesthesiology and Reanimation, Istanbul, Turkey; 7 Department of Medicine–DIMED, University of Padua, Padua, Italy; 8 Institute of Anesthesia, University Hospital of Padua, Padua, Italy; 9 Department of Anesthesiology and Reanimation, Ataturk University School of Medicine, Erzurum, Turkey; 10 Anesthesia and Intensive Care Unit, University-Hospital of Padova, Padua, Italy

To the Editor,

The transversalis fascia lies deep to the transversus abdominis muscle (TAM) and extends around the quadratus lumborum (QL) muscle beyond its lateral border [1, 2]. The transversalis fascia plane block (TFPB) is performed by injecting a local anesthetic between the transversalis fascia and TAM [2], and its clinical efficacy is well supported by the literature [3–6]. However, even if some histological studies evaluated the transversalis fascia [7, 8], there is no anatomical study on the macroscopic spread of this block [1, 9]. This study aimed to evaluate the macroscopic spread of TFPB using different volumes in a cadaveric model.

Ethical approval was obtained from the Istanbul Medipol University Ethics and Research Committee (30.10.2025, No.1281). A fresh-frozen 56-year-old female cadaver with no previous abdominal surgery, trauma, or pathological changes involving the lower abdominal region was included. The block was performed in the supine position with a low-frequency (2–6 MHz) convex transducer (Clarius, Canada) and a 22-G × 100 mm peripheral nerve block needle (Stimuplex® Ultra 360®, B-Braun, USA). The transducer was placed transversely between the iliac crest and costal margin. The external oblique, internal oblique, and TAM muscles were visualized. The transducer was then advanced posteriorly to visualize the insertion of the TAM and its aponeurosis. The needle was inserted through the muscles into the perirenal fat tissue deep to the TAM, surrounded by the transversalis fascia, using an in-plane technique and an anterior–posterior trajectory. A methylene blue solution was injected (30 ml on one side and 40 ml on the other). The dissection technique is described in Supplementary material 1.

There was staining anterior to the QL muscle, of the ilioinguinal-iliohypogastric nerves, of the subcostal nerve (T12), and of the paravertebral space between the 11th and 12th ribs on both sides, and when the dissection was extended cranially, there was bilateral staining of the 11th intercostal nerve. However, no staining was observed in the lumbar plexus on either side (Fig. 1).

**Fig. 1:**
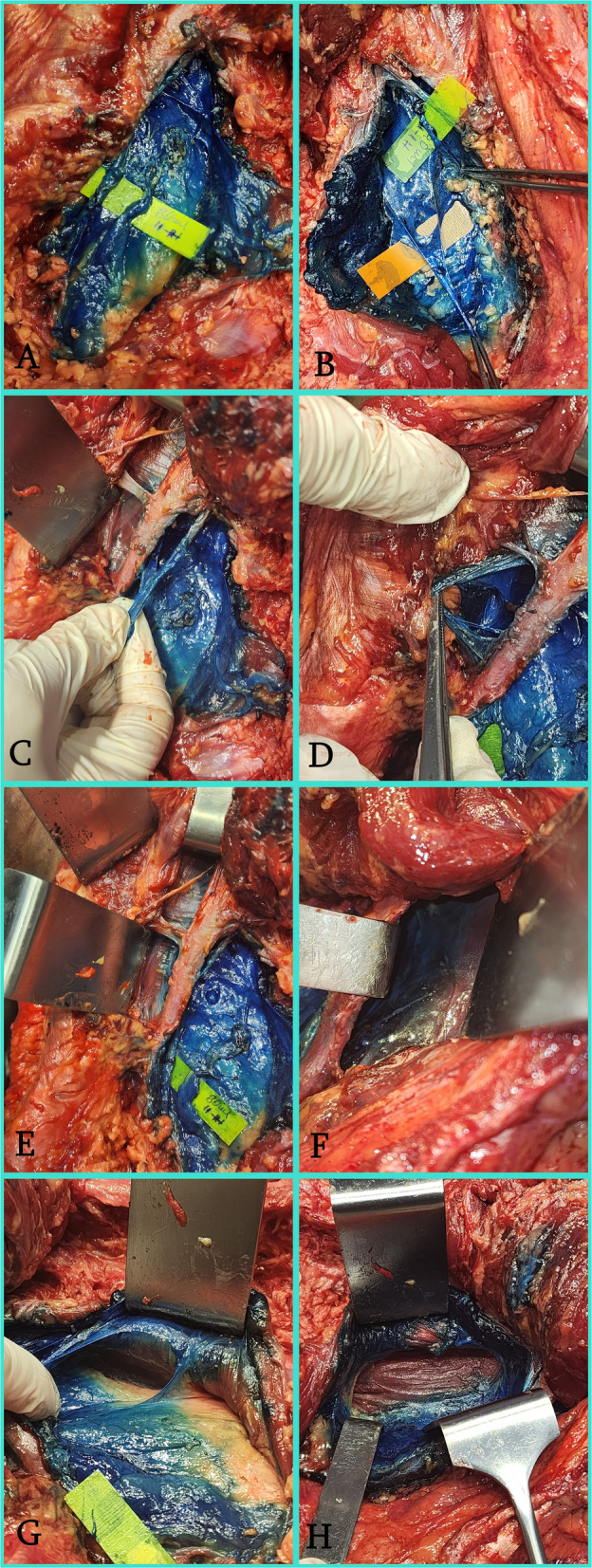
Macroscopic dissection findings comparing dye spread following ultrasound-guided TFPB with 30 mL and 40 mL. **A** The 30 mL injection side demonstrated intense staining of the ilioinguinal and iliohypogastric nerves (marked with yellow labels). **B** The 40 mL injection side showed similar intense staining of the ilioinguinal and iliohypogastric nerves. **C** Demonstration of the subcostal nerve (T12) successfully stained with dye on the 30 mL side. **D** Cranial spread reaching and staining the 11th intercostal nerve on the 30 mL side. **E** Dye spread visualized extending above the 11th and 12th ribs on the 30 mL side. **F** Intense dye accumulation was observed in the paravertebral region between the 11th and 12th ribs on the 40 mL side, indicating more extensive posterior spread. **G** Deep dissection of the 30 mL side revealed no dye staining in the lumbar plexus, confirming the motor-sparing characteristic of the block. **H** Deep dissection of the 40 mL side, similarly showing the absence of dye in the lumbar plexus. *(II: Ilioinguinal nerve; IH: Iliohypogastric nerve)*

Our study shows that TFPB preserves the lumbar plexus, involves the subcostal, ilioinguinal, and iliohypogastric nerves, and has paravertebral spread. We found no difference in staining between the 30 mL and 40 mL injections; therefore, we speculate that 30 mL may be sufficient to achieve the observed staining. Paravertebral spread was observed at both volumes, more pronounced at 40 mL, and demonstrates the clear and consistent efficacy of the block [10].

This study has several limitations. First, it was performed on a single cadaver, which limits generalizability. Second, the cadaveric models and dye used cannot fully replicate in vivo conditions. Finally, although macroscopic spread was evaluated, functional correlates such as sensory blockade and dermatome distribution could not be assessed.

Future studies can be based on these anatomical foundations, and contributions to the literature can be made by conducting dermatome mapping studies evaluating different volumes.

## Supplementary Information

Supplementary Material 1. Dissection technique.

## Data Availability

All the data used to prepare this manuscript are presented in the manuscript.
