# Supplementary material for: Spread of injectate during the transversalis fascia plane block: a preliminary anatomical investigation
Source: J Anesth Analg Crit Care. 2026 Mar 18;6:45. doi: 10.1186/s44158-026-00375-x (PMC12998043; doi:10.1186/s44158-026-00375-x)
Supplement: Supplementary file 1 — Supplementary Material 1. Dissection technique. [file 44158_2026_375_MOESM1_ESM.docx]

**Spread of Injectate During the Transversalis Fascia Plane Block: An Anatomical Investigation**

**– Supplementary Material –**

For dissection, a transverse skin incision was made along the 12th rib extending laterally, a longitudinal incision was performed along the line of the vertebral spinous processes in the craniocaudal direction, and an additional incision was made following the iliac crest. After removal of the skin and subcutaneous adipose tissue, the thoracolumbar fascia and the latissimus dorsi muscle were dissected from their attachments to the vertebral spinous processes and displaced laterally. To achieve complete visualization of the QL muscle, the erector spinae muscle group was dissected transversely from its medial attachment points on the spinous processes of the vertebrae and elevated in a craniocaudal direction using a retractor. At this stage, the multifidus muscle was preserved in the medial plane, and the dissection was continued laterally. Advancing the dissection line between the iliac crest and the 12th rib allowed access to the lateral and anterior surfaces of the quadratus lumborum muscle, and the plane of the TF was clearly identified. Within this plane, the T12-derived subcostal nerve and the L1-derived iliohypogastric and ilioinguinal nerves were localized bilaterally.
